# Gene-chip studies of adipogenesis-regulated microRNAs in mouse primary adipocytes and human obesity

**DOI:** 10.1186/1472-6823-11-7

**Published:** 2011-03-22

**Authors:** Pernille Keller, Valentina Gburcik, Natasa Petrovic, Iain J Gallagher, Jan Nedergaard, Barbara Cannon, James A Timmons

**Affiliations:** 1 Royal Veterinary College, University of London, UK; 2 Department of Physiology, The Wenner-Gren Institute, Stockholm University, Sweden; 3 Tissue Injury & Repair Group, The Chancellor's Building, University of Edinburgh, EH16 4SB, UK; 4 Department of Molecular Genetics, Novo Nordisk A/S, DK-2760 Måløv Denmark

**Keywords:** primary white and brown adipocytes, microRNAs, microarray, EXIQON, Affymetrix, Adipose tissue: adipocyte, transcriptome

## Abstract

**Background:**

Adipose tissue abundance relies partly on the factors that regulate adipogenesis, i.e. proliferation and differentiation of adipocytes. While components of the transcriptional program that initiates adipogenesis is well-known, the importance of microRNAs in adipogenesis is less well studied. We thus set out to investigate whether miRNAs would be actively modulated during adipogenesis and obesity.

**Methods:**

Several models exist to study adipogenesis *in vitro*, of which the cell line 3T3-L1 is the most well known, albeit not the most physiologically appropriate. Thus, as an alternative, we produced EXIQON microarray of brown and white *primary *murine adipocytes (prior to and following differentiation) to yield global profiles of miRNAs.

**Results:**

We found 65 miRNAs regulated during *in vitro *adipogenesis in primary adipocytes. We evaluated the similarity of our responses to those found in non-primary cell models, through literature data-mining. When comparing primary adipocyte profiles, with those of cell lines reported in the literature, we found a high degree of difference in 'adipogenesis' regulated miRNAs suggesting that the model systems may not be accurately representing adipogenesis. The expression of 10 adipogenesis-regulated miRNAs were studied using real-time qPCR and then we selected 5 miRNAs, that showed robust expression, were profiled in subcutaneous adipose tissue obtained from 20 humans with a range of body mass indices (BMI, range = 21-48, and all samples have U133+2 Affymetrix profiles provided). Of the miRNAs tested, mir-21 was robustly expressed in human adipose tissue and positively correlated with BMI (R2 = 0.49, p < 0.001).

**Conclusion:**

In conclusion, we provide a preliminary analysis of miRNAs associated with primary cell *in vitro *adipogenesis and demonstrate that the inflammation-associated miRNA, mir-21 is up-regulated in subcutaneous adipose tissue in human obesity. Further, we provide a novel transcriptomics database of EXIQON and Affymetrix adipocyte profiles to facilitate data mining.

## Background

Obesity is a major global health problem linked to serious medical conditions including diabetes, heart disease, arthritis and cancer [1]. Adipose tissue is not only a main site of energy storage but also an important endocrine organ. It is a crucial regulator of energy balance and glucose homeostasis in mammals (reviewed in [2]). Imbalances in energy homeostasis cause obesity. Most of the lipid reserves in the human body are stored in white adipose tissue (WAT) that predominates in adult humans. Brown adipose tissue (BAT), despite its ability to accumulate lipids, has a role not in storing but in converting fat into heat. Uncoupling Protein 1 (UCP-1) in the inner membrane of brown-fat mitochondria uncouples electron transport from ATP production, allowing energy dissipation, thus helping to regulate body temperature [3]. Recent evidence indicates that brown fat is important not only in newborns as previously thought but also in adult humans [4]. It has been suggested recently that human obesity is associated with altered function of brown adipose tissue [5,6] or appearance of brown adipocytes within WAT [7]. Understanding the regulation of the pathways that lead to proliferation and differentiation of white and brown pre-adipocytes could be crucial for revealing the underlying mechanisms of obesity. While primary white and brown pre-adipocytes look identical, these two cell types originate from separate precursor cells [8] in the early embryo [9,10]. Brown fat cells arise from a pre-muscle cell lineage [8]. In addition to the classic brown adipocytes, a different type of brown fat cells seems to exist in tissues where WAT predominates. These cells are more closely related to white adipocytes but have the potential to induce UCP1 expression [11]. It has been suggested that adipogenesis is regulated by PPARβ/δ followed by PPARγ and C/EBPα promoting differentiation into mature adipocytes [12]. Maturation of BAT and WAT follow a similar adipogenic transcriptional program, albeit several genes show cell type-dependent expression. For instance, Prdm16 is expressed in BAT and when ectopically expressed in white preadipocytes, can promote brown fat differentiation [13].

A growing body of evidence implicates a role for microRNAs (miRNAs) in adipogenesis and obesity [14,15]. Indeed, the lineage difference between white and brown adipocytes has not only been shown on the basis of different mRNA expression but also at the levels of microRNA expression, as shown by Walden et al. [16]. Further, miRNAs can both target [17] and be induced by transcription factors [18]. MiRNAs are short non-coding RNA molecules that allow for fine-tuning of protein expression *in vivo *[19]. They act at a post-transcriptional level by binding to the 3' untranslated region (UTR) of an mRNA largely leading to translational regulation *in vivo *in mammals [19,20]. There are several examples of miRNAs that regulate metabolic processes associated with metabolic disease. Mir-122 can regulate lipid/glucose homeostasis in the liver [21]. Mir-375 [22] and miR-9 [23] can influence insulin secretion, while mir-34a can increase hormone secretion in insulin-secreting cells when exposed to palmitate [24]. The first miRNA found to play a role in maturation of human adipocytes was miR-143 [25,26]. miR-143 and also miR-103 can induce adipogenesis in 3T3-L1 adipocytes and augment or accelerate expression of several key adipogenesis-regulated genes, such as FABP4 and adiponectin [27]. Activation of the miRNA cluster miR-17~92 enhances adipogenesis [28] while let-7 and miR-27 impair adipogenic differentiation [29,30]. Several miRNAs are upregulated in adipose tissue in animal models of metabolic disease and/or obesity, e.g. mir-125a [31], mir29 [32], and mir-143 [33], while adipose tissue miR-519d expression is associated with human obesity [15]. Recently, Ortega et al. claimed that the expression of 70 miRNAs is modulated during differentiation of human adipocytes, with 15 of them showing dysregulation in obese individuals compared with non-obese persons [14].

Expression profiling of miRNAs in various adipocyte culture systems has yielded inconsistent results so far [25-27]. Studies have mostly been performed using cell lines and thus there is a lack of knowledge of the miRNA expression profile in primary adipocyte cells. Furthermore, there is no parallel analysis of both white and brown differentiating primary adipocytes. Thus, we performed a global expression profiling of miRNAs in white and brown murine primary cell cultures before and after differentiation. We found that 51 unique miRNAs were significantly upregulated and 14 significantly downregulated after the differentiation into adipocytes. We also demonstrated that differences in miRNA regulation between white and brown adipocytes during maturation are surprisingly modest. We investigated the expression of selected maturation-associated miRNAs in subcutaneous adipose tissue from humans with varying body mass indices and found that mir-21 expression correlated positively with human obesity. The present manuscript presents these miRNA gene-chip data sets, along with a pilot Affymetrix data set of n = 33 human adipose global U133+2 tissue profiles, as a novel obesity and adipogenesis database for others to utilise.

## Methods

### Murine samples

The experiments were approved by the ethical committee of North Stockholm. Male outbred NMRI mice, 4 weeks old, were used for the preparation of primary cultures of brown and white preadipocytes, as described previously [11,34]. Mouse white adipose tissue (WAT) was harvested from the epididymal fat pad, while mouse brown adipose tissue (BAT) was harvested from the interscapular region. Preadipocytes were differentiated for 3 days (preadipocyte population) and 7 days (mature adipocyte population) *in vitro*. The culture medium was Dulbecco's modified Eagle's medium with 10% (v/v) newborn calf serum (Invitrogen), 2.4 nm insulin, 25 μg/ml sodium ascorbate, 10 mm HEPES, 4 mm glutamine, 50 units/ml penicillin, and 50 μg/ml streptomycin. The cells were grown at 37°C in an atmosphere of 8% CO_2 _in air with 80% humidity. The medium was changed on day 1 and then every second day. RNA was extracted from preadipocytes and adipocytes using Trizol (Invitrogen), according to the manufacturer's instructions.

### Human samples and physiological measurements

Twenty healthy subjects of Scandinavian origin with normal glucose tolerance but different degrees of obesity participated in the study conducted by CIM, Copenhagen. The study included individuals from a previous analysis [35] and individual data can be found along with global transcript profiles at GSE27951. Their mean (SD) age, BMI and VO_2max _were 46 (13) years, 32 (7) kg/m^2 ^and 31 (13) VO_2max_/kg, with the range in BMI being 21-48 kg/m^2^. Adipose tissue biopsies were obtained from the subcutaneous abdominal region (from the area below the umbilicus in a 4-cm range on either side) using the percutaneous needle biopsy technique with suction. Biopsies were quickly dissected free from visible blood and connective tissue and frozen in liquid nitrogen. To avoid interference of circadian rhythms and post-prandial responses, biopsies were obtained between 8 am and 10 am following an overnight fast. A general health examination and an oral glucose tolerance test (OGTT) were performed as previously described [35]. All tissue processing and RNA work was done independently of CIM, Copenhagen and CIM had no input on data analysis.

### RNA extraction and quality

Frozen tissue was homogenised in Trizol™ (Invitrogen) using a motor-driven homogenizer (Polytron, Kinematica, Newark, NY, USA). RNA was extracted according to manufacturer's protocol, as previously described [35]. The quality of the total RNA was examined on an Agilent Bioanalyzer. Human adipose tissue RNA for miRNA Taqman qPCR assays was of good quality with spectrophotometer readings of A260/280 >1.7 and RIN scores >7.5. Murine RNA had A260/280 >1.9 and RIN scores >8.

### Exiqon miRCURY™ miRNA arrays

Global microRNA expression analysis was performed using Exiqon (Vedbæk, Denmark) pre-spotted miRCURY™ V8.1 LNA microRNA microarrays. Preadipocyte and mature adipocyte samples (n = 3+3, for brown adipocytes and n = 3+3 for white adipocytes) were run individually on the arrays, i.e. cell cultures were not pooled. The arrays consist of 1458 capture probes (or as annotated on the GEO files) that are enhanced using locked nucleic acid (LNA) technology to normalize the Tm of the capture probes, as insertion of 1 LNA molecule into the capture probes increases the Tm by 2-8°C. Total RNA was labelled with Hy^3^-dye using Exiqon's miRCURY LNA array labelling kit (Vedbæk, Denmark) with the inclusion of the miRCURY LNA array synthetic spike-in miRNAs in the labelling reaction. Two μg of total RNA was incubated with the Hy^3 ^dye, labelling enzyme and spike-in microRNAs for 1 hour at 0°C. The enzyme was then heat-inactivated at 65°C for 15 minutes, followed by addition of 2 x hybridisation buffer. The samples were incubated at 95°C for 5 minutes, protected from light. The samples were then briefly spun down and filtered through a 0.45-micron durapore filter (Millipore), and 40 μl sample was loaded to the arrays by capillary force using a cover slip (Erie Scientific). The arrays were incubated at 60°C for 16 hours, then washed briefly in 60°C wash buffer A, rinsed in wash buffer B followed by a 2-minute wash in wash buffer B and a 2-minute wash in wash buffer C, according to the manufacturer's instructions. For drying the arrays, they were spun for 2 minutes at 1000 rpm followed by immediate scanning using an Agilent G2565BA microarray scanner. Data were analyzed by QuantArray^® ^software using the fixed-circle approach. Background subtracted signal intensities were normalized in R using quantile normalization. Data were then analysed using SAM analysis as previously described [19]. In this case on relevant murine probes and signals above background were utilised in the SAM analysis. The analysis to determine adipogenesis related miRNAs was performed using brown-cell and white-cell gene chips combined, to increase the sample size and hence statistical power. A selected subset of miRNAs modulated more than 1.5-fold was selected for further analysis by real-time qPCR.

### qPCR for the detection of microRNA expression levels in adipocytes

The expression of 10 miRNAs that showed differential expression on the microarrays were measured in 12 RNA samples (3 preadipocyte and 3 adipocyte samples from both brown and white fat) using the Taqman^® ^MicroRNA assays (Applied Bioystems) that detect mature miRNA. 10 ng of total RNA was reverse transcribed using the microRNA-specific looped primer and the TaqMan^® ^MicroRNA Reverse Transcription Kit (Applied Biosystems, PN 4366597), according to manufacturer's instructions. Thermal cycling conditions were as follows: 16°C for 30 min, 42°C for 30 min, and 85°C for 5 min. For qPCR, the TaqMan^® ^2X Universal PCR Master Mix, No AmpErase^® ^UNG was used (Applied Biosystems, PN 4324020). Experiments were performed in triplicates on a 7900HT Fast Real-Time PCR System (Applied Biosystems), 9600 emulation mode. The Real-Time cycling conditions were: 95°C for 10 minutes followed by 50 cycles of 95°C for 15 sec and 60°C for 1 min. The expression of the reference RNAs used for normalisation, snoRNA142 (murine samples) and RNU48 (human samples), did not show variation between samples or subjects. All reactions were run single-plex and analysed and quantified using the ΔΔCt method. Data are expressed as a fold change from the value in non-obese persons for the human samples or as a fold change from preadipocyte-levels for the murine samples. miRNA data obtained by qPCR were analysed using a t-test to compare differences between ΔCt values (Ct value of target miRNA minus Ct value of reference RNA) either between obese and non-obese persons, or between adipocytes and preadipocytes. For all analyses, *P *< 0.05 was considered significant.

### qPCR for the detection of microRNA expression levels in human fat

Of the 10 miRNAs that showed expression in the adipocyte cultures, we chose a subset of 5 miRNAs (mir-34c, mir-143, mir-24, mir-720 and mir-21) to measure in human adipose tissue RNA samples from obese persons (BMI >30, n = 10) and non-obese persons (BMI <30, n = 10). The phenotype of these subjects has been described elsewhere [7,19] and the subject demographics can be found, along with global mRNA transcriptome data at GSE27951. This data represents a large pilot study of n = 33 human adipose tissue samples. We have previously reported selected gene changes in this tissue cohort. The RNA profile of the tissue did not support the conclusion that metabolic or mitochondrial genes were down-regulated in human obese adipose tissue and thus contrasts with Dahlman et al [36]. Dahlman et al did not release the CEL files so we cannot determine if this difference is biological or technical in nature. Using RNA isolated from our adipose tissue samples (10 ng total RNA) the RNA was reverse-transcribed using reverse transcription reagents (Applied Biosystems), according to the manufacturer's protocol to profile microRNAs.

Detection of cDNAs was performed using an ABI-PRISM^® ^7900 Sequence Detection system (Applied Biosystems). Primers (Invitrogen) were designed by Primer Express software (Applied Biosystems) or by the Universal Probe Library (Roche Applied Science). A pre-optimized primer and probe assay for 18S rRNA was used as an endogenous control (Applied Biosystems). Primers and probes were pre-mixed with TaqMan Universal Master Mix or SYBR^®^GREEN PCR Master Mix (Applied Biosystems) and applied to 384-well MicroAmp Optical barcode plates (Applied Biosystems). cDNA aliquots of 4 μl were added in triplicates. The amplification of genomic DNA typically amounted to a maximum of <1% of the target gene when extracting RNA by TRIzol [37]. Thermal cycling conditions were: 2 min at 50°C, 10 min at 95°C and 40 cycles of 15 s at 95°C and 1 min at 65°C. Two-fold dilutions series were performed for all target genes and endogenous controls to determine the amplification efficiency.

### Gene Ontology analysis using EASE

A miRNA binds to the 3' UTR of a target mRNA via the "seed" region of the mature miRNA (nt 2-7). For mir-21, we used Gene Ontology (GO) analysis to obtain an overview of the main classes of biological functions of genes predicted to be targets for this miRNA. For miRNA target prediction categorised by Gene Ontology, we used EASE (version 2.0) and a predicted list of conserved miRNA target genes obtained from Targetscan (http://www.targetscan.org/, release 5.0). For the EASE analysis, we enlisted a false discovery rate (FDR) and an EASE score, which we calculated using 500 permutations/iterations. It should be noted that mir-21 is unlikely to be the only obesity regulated miRNA and as such this ontology analysis should be considered hypothesis generating rather than definitive.

### Network analysis using PubGene

The connectivity of putative miRNA targets was analysed using the literature co-citation network PubGene (http://www.pubgene.org). The following explanation is extracted from the PubGene Public Service User Guide. PubGene lists records that co-cite gene identifiers. Co-citation suggests biological relationship between the implicated genes. The Bio Networks tool presents related genes graphically as networks. A count is made of the MEDLINE records where both the query gene and a neighbour appear (or a recognized synonym of either of these). Neighbours of a gene are thereby ranked according to this count. The Bio Associations tool prepares lists of keywords associated with gene or protein terms. Using this tool, we performed a search for process-association with an individual gene ID. The Bio Associations tool returns a list of GO terms for process-associations that co-occur with the query gene in Medline records. The list is ranked depending on scores assigned to each of the co-occurring terms. Scoring was computed by a probabilistic method. Using this method, scores approaching 0 show the strongest association between the query gene and GO term. In principle, scores can be in the range of 0 to 1. The published formula shows the probability of the term and the gene occurring together the observed number of times under the assumption that their co-occurrence is not meaningful but instead simply a random event (i.e. the probability of the null hypothesis). We utilised this approach as a descriptive tool.

## Results

### Identification of adipogenesis-regulated miRNAs in primary white and brown murine adipocytes using miRNA arrays

**Figure 1:**
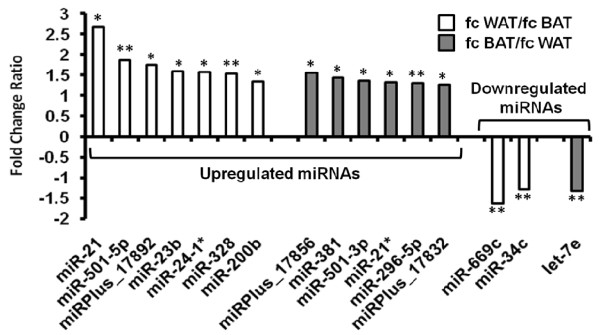
**miRNA regulation during adipocyte differentiation**. Differences between miRNA regulation during differentiation between white and brown adipocytes are shown as a fold difference (ratio) of fold changes. Student T-test was performed to calculate the significance in these differences. * depicts p < 0.1, ** depicts p < 0.05.

To examine the role of the post-transcriptional regulators of adipogenesis in a physiologically relevant model, we profiled primary white and brown pre-adipocytes before and after differentiation into mature adipocytes (n = 3+3 for each tissue type) using Exiqon miRNA arrays, v.8.1. Data sets were highly consistent both after background subtraction and after quantile normalisation of the background subtracted data. The analysis was performed in two ways. Since there were no large differences between miRNA expression in brown and white adipocytes, we used the data from both cell types combined, to increase the sample size and hence statistical power (Additional file 1). 51 unique miRNAs were significantly upregulated and 14 miRNAs were downregulated during the differentiation of both white and brown preadipocytes (FDR <10%). Then we did the analysis of the significant data from brown and white cells separately using a standard Student T-test to look for the differences in miRNA regulation during white and brown adipocyte maturation. While this clearly has limitations, six miRNAs showed a trend for a stronger upregulation during white pre-adipocyte differentiation compared with brown pre-adipocytes, while another 6 miRNAs showed a trend for a stronger upregulation during brown pre-adipocyte development (see Figure 1). Only two miRNAs seemed to be downregulated more strongly during white pre-adipocyte maturation compared to brown, while only one miRNA showed a trend for a stronger downregulation during brown adipocyte differentiation. Such trends are interesting but require confirmation.

In general, differences in miRNA regulation between white and brown adipocytes during maturation are not pronounced. The six miRNAs tending to demonstrate a stronger upregulation during the white adipocyte differentiation included miR-24-1* and miR-23b, members of a recently identified miR-23b cluster. Another miRNA that seemed more strongly upregulated during the white adipocyte differentiation was miR-21. This upregulation was not significant in the main analysis (FDR >10%), but we chose to follow up on this miRNA in later experiments because of its reported role in proliferation [38] and inflammation [39]. We also decided to follow up on miR-143, which we found upregulated in both white and brown adipocytes, as mir-143 is induced upon differentiation of human preadipocytes and 3T3-L1 fibroblasts [25-27].

Six miRNAs showed a trend for a stronger upregulation during the brown adipocyte differentiation - miRPlus_17856, mmu-miR-381, mmu-miR-501-3p, mmu-miR-21*, mmu-miR-296-5p and miRPlus_17832. In contrast to the stronger upregulation of miR-21 in white cells, miR-21* (the opposite arm of miR-21) seemed to be more strongly upregulated in brown cells. Of the 14 downregulated miRNAs, miR-669c and miR-34c, tended to be downregulated further during the maturation of white adipocytes compared to browns. One miRNA, mmu-let-7e, seemed more downregulated during the brown adipocyte maturation. Critically, no miRNA showed expression only in browns or whites in this gene chip study, nor did we see an opposite regulation pattern in the two cell types, suggesting that at this level of sensitivity, we captured adipogenesis-regulated miRNAs, rather than miRNAs involved in cell lineage determination.

### qPCR profiling of miRNAs in primary white and brown adipocyte cultures

**Figure 2:**
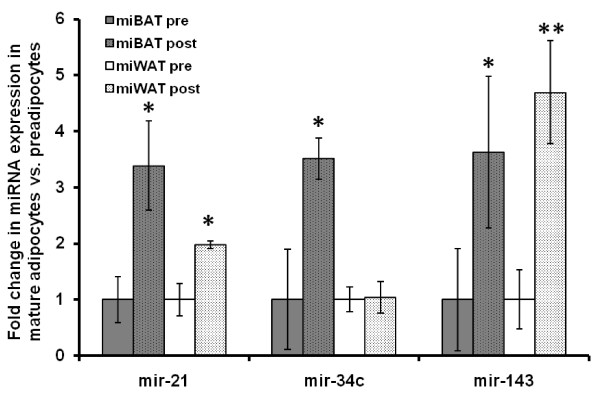
**Expression levels of mir-21, mir-34c and mir-143 in primary murine brown and white preadipocytes and mature adipocytes (n = 3+3 for each tissue)**. miRNA expression levels are shown as fold change from their respective preadipocyte level and normalised to the small nucleolar RNA snoRNA142. * depicts p < 0.05, ** depicts p < 0.01.

From the list of the miRNAs that were differentially expressed during adipogenesis of primary white or brown murine adipocytes, we selected a subset of 10 miRNAs for profiling using Taqman-based qPCR. Of these 10 miRNAs, only mir-21, mir34c and mir-143 were differentially expressed between mature adipocytes and preadipocytes (p < 0.05), see Table 1 and Figure 2. Mir-34c upregulation during brown adipocyte maturation was in contrast to our microarray data and this result must be treated with caution. When comparing the EXIQON chip and the TaqMan assay, mir-34c demonstrated the opposite result suggesting that either the gene-chip probe or the TaqMan assay is unreliable. Mir-136 and mir-718 were not detectable in the adipocyte cultures using the Taqman assays-on-demand, while mir-346, mir-298, mir-330 and mir-501 were expressed at low levels (Ct levels above 33), see Table 1. This suggests that currently there is no gold standard method (when RNA is limiting) to validate miRNA data profiles. It should however be noted that the development of the EXIQON chip has progressed since Version 8 and some of these issues may now be resolved. Nevertheless, the miRNAs mir-34c, mir-143, mir-24, mir-720 and mir-21 showed robust expression in the adipocyte cultures, and these 5 miRNAs were thus profiled in subcutaneous adipose tissue from healthy humans with different BMIs to examine their regulation in adipose tissue expansion.

**Table 1:** MicroRNA Ct levels ± standard deviation of samples (n = 3)

miRNA	WAT preadipocytes	WAT adipocytes	BAT preadipocytes	BAT adipocytes
miR-21	23.2 ±0.4	22.3 ±0.2	23.9 ± 0.8	22.2 ±0.2
miR-24	25.5 ±0.5	25.2 ±0.1	25.5 ±0.7	24.9 ±0.3
miR-34c	28.8 ±0.1	28.7 ±0.4	29.0 ±1.2	27.1 ±0.2
miR-136	ND	ND	ND	ND
miR-143	31.6 ±0.6	29.4 ±0.6	31.8 ±1.2	29.3 ±0.5
*miR-298*	*33.2 ±0.2*	*33.8 ±0.2*	*33.5 ±0.6*	*33.5 ±0.4*
*miR-346*	*35.7 ±1.1*	*36.4 ±0.8*	*35.7 ±0.6*	*34.7 ±0.4*
*miR-501*	*34.5 ±0.9*	*34.4 ±0.9*	*34.6 ±0.1*	*34.4 ±0.4*
miR-718	ND	ND	ND	ND
miR-720	22.5 ±0.3	22.9 ±0.1	23.4 ±0.4	22.9 ±0.7

All miRNA assays are pre-developed assays (PDA) from Applied Biosystems. Reference RNAs used for normalisation, snoRNA142, did not show variation between samples. ND: Not detectable. Note that miRNA expression values where Ct = >>30 could indicate that the TaqMan assay is non-functional.

### A weighted context score ranking analysis of the global changes in adipogenesis-induced changes in miRNA expression

We previously validated a novel ranking system to calculate the collective impact of both upregulated and downregulated miRNAs *in vivo *[19]. We combined the conserved target site criteria from the Targetscan database with cell-specific mRNA expression data [8] and generated a list of genes reliably expressed in adipocytes and that would be potentially regulated by the combined action of all the changing miRNAs. We combined the two non-overlapping lists of up-regulated and down-regulated putative targets of miRNAs as previously described [19] and tested for the targeted biological processes using the ontological analysis (Additional file 2) using the global adipocyte transcriptome as a back-ground. Highly significant enrichment was uniquely found within the first quartile of ranked genes, including transcription regulation (p < 1.62 × 10^-6^) and regulation of cellular metabolic processes (p < 4.3 × 10^-4^). The fourth quartile of conserved wCCS targets did not show such enrichment and acts as the control list for this validated method [19]. Ontological enrichment of target genes shows in a statistically significant manner that distinct biological processes are targeted, notably the ones related to transcription and metabolism even when using only expressed miRNA predicted gene targets (a gene list that has an ontological bias *per se*).

### Network analysis of putative miRNA targets

**Figure 3:**
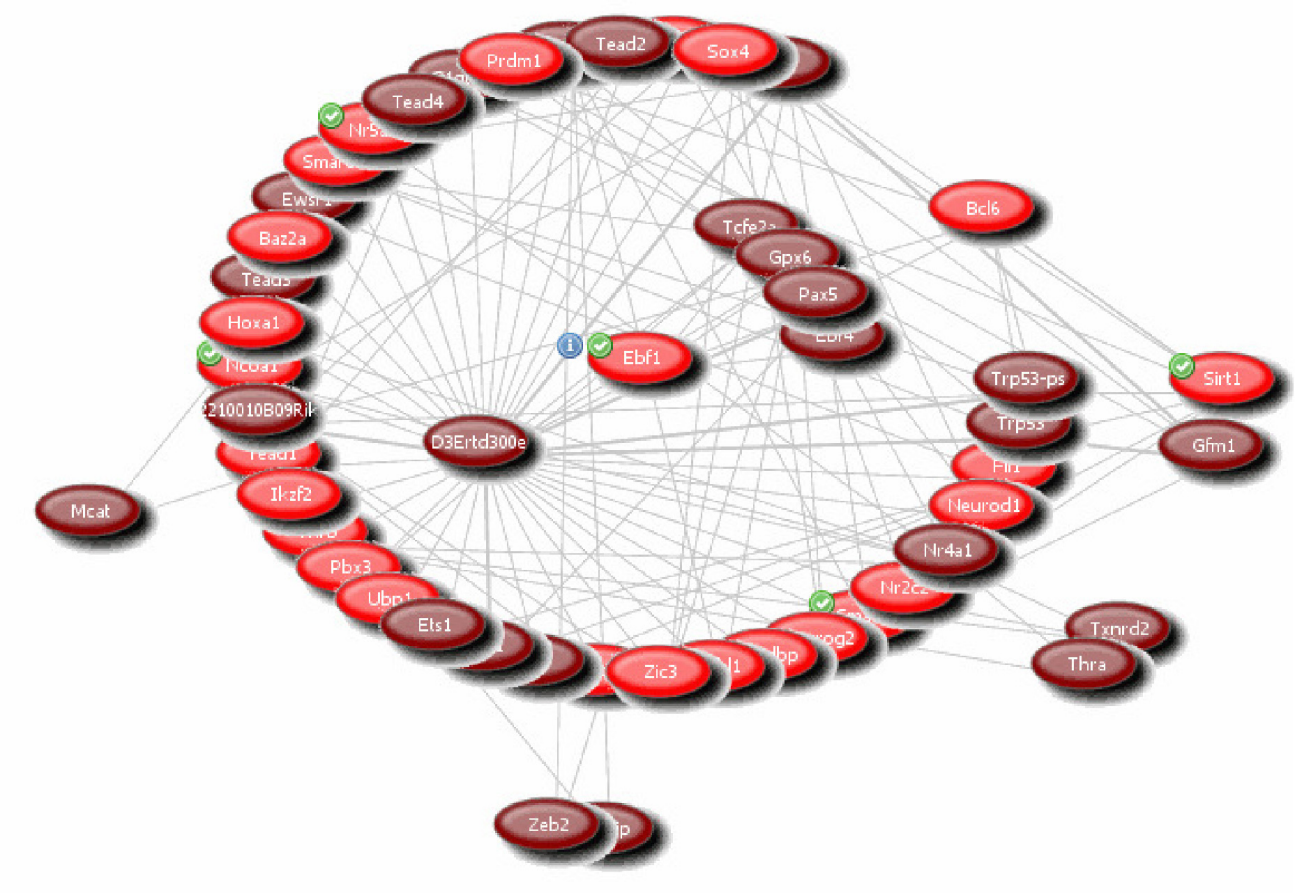
**Network showing the interconnections between 1q miRNA targets belonging to GO category "SP_PIR_Transcription Regulation genes" (colored in red) and their first order literature neighbours (colored in brown)**. The connectivity of putative miRNA targets was analysed using the literature co-citation network PubGene (http://www.pubgene.org), see Materials and Methods.

The connectivity of first quartile ranked genes belonging to the ontology category SP_PIR_Transcription Regulation genes (FDR <2 × 10^-6^, in total 37 genes) was analysed using the literature co-citation network PubGene (http://www.pubgene.org). PubGene lists records that co-cite gene identifiers. Co-citation suggests biological relationship between the implicated genes. The network showing the interconnections between analysed genes and their first order literature neighbours is shown in Figure 3. In total there are 132 connections, while the network based on probability of chance co-occurrence contains 67 connections. The most connected gene from this analysed group was Sirt1, which linked directly to 5 other genes from the analysed group (Sox4, Bcl6, Neurod1 and Smad7). Using PubGene process associations, we annotated all 37 genes to biological processes by probabilistic scoring. Five out of 37 analysed genes (Ebf1, Ncoa1, Nr5a2, Sirt1 and Smad7) are involved in fat cell differentiation (p < 1.9 × 10^-7^) (see Additional file 1), while all of the 37 genes play a role in differentiation, development and/or proliferation processes (p < 0.001). PubGene associated are not fully validated and such association should be taken as hypothesis generating.

### Regulation of adipogenesis-regulated miRNAs in obesity

**Figure 4:**
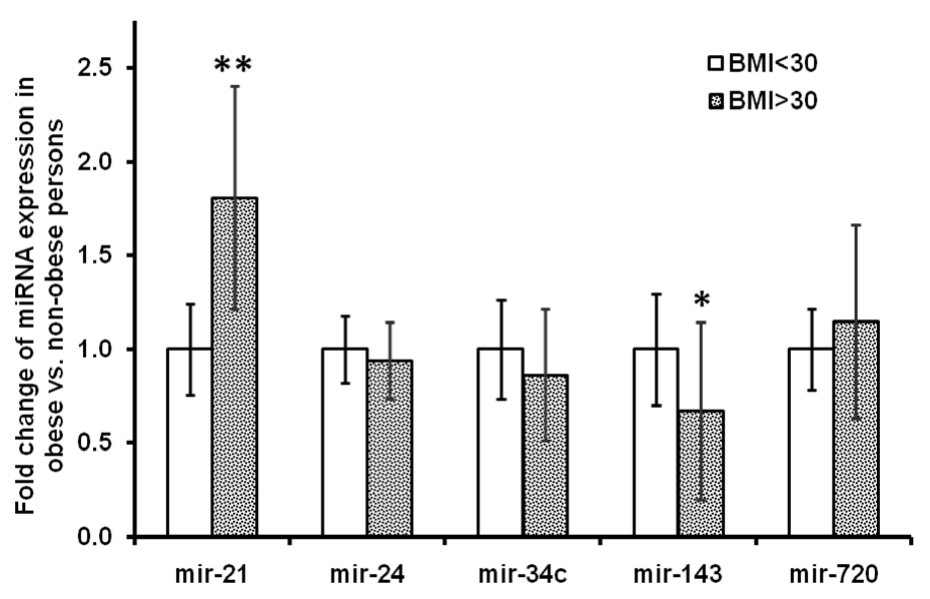
**Expression levels of mir-21, mir-24, mir-34c, mir-143 and mir-720 were measured in subcutaneous adipose tissue of obese (BMI >30, n = 10) and non-obese (BMI <30, n = 10) healthy persons**. miRNA expression levels are normalised to RNU48 and shown as fold change from the expression level of the non-obese persons. * depicts p < 0.05, ** depicts p < 0.001.

**Figure 5:**
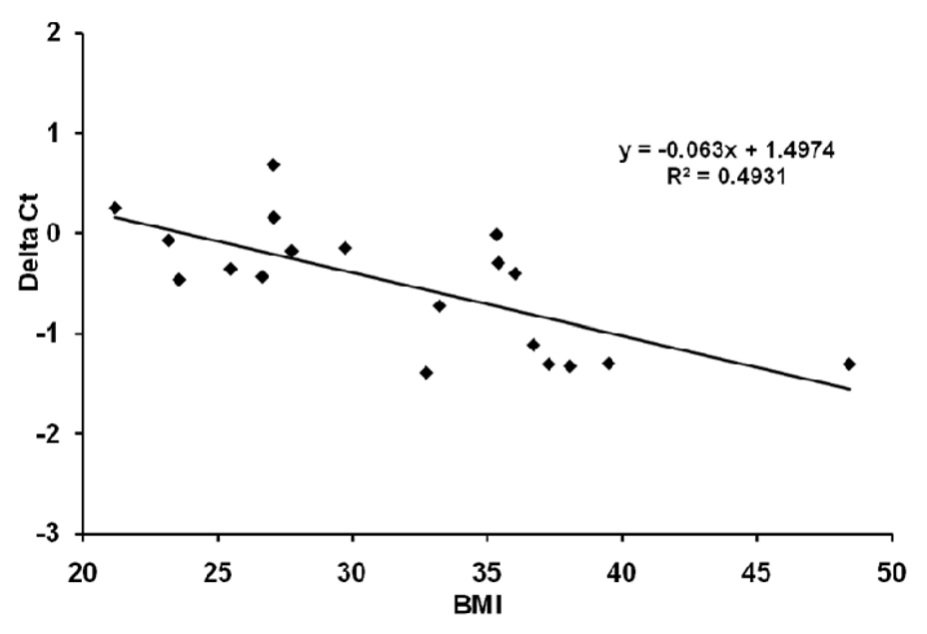
**Regression plot of mir-21 expression levels in subcutaneous adipose tissue of healthy persons (n = 20) with a range in BMI (21-48)**. Data are presented as ΔCt levels (difference between Ct value for miR-21 and Ct value for control RNU48) versus BMI and thus represents a positive correlation between mir-21 expression levels and BMI, p < 0.001.

Five miRNAs (mir-21, mir-143, mir-34c, mir-24 and mir-720) were profiled in subcutaneous adipose tissue from healthy humans with varying degrees of obesity. Mir-21 showed higher expression in persons with a BMI >30, while mir-143 showed lower expression in persons with a BMI >30 (p < 0.05), see Figure 4. There was a strong positive correlation of mir-21 expression in human adipose tissue with BMI (p < 0.001, see Figure 5).

## Discussion

We performed global expression profiling of miRNAs before and after the differentiation of primary murine white and brown preadipocytes into mature adipocytes, in order to discover post-transcriptional regulators of adipogenesis. We found 65 miRNAs differentially expressed following differentiation of preadipocytes into adipocytes, but only subtle differences in fold-change of miRNAs between white and brown adipocytes, suggesting that the identified miRNAs most likely play a role in adipogenesis *per se *rather than in cell lineage determination. Ontological analysis of the target genes of the miRNAs regulated during *in vitro *adipogenesis indicated that processes related to transcription and metabolism are potentially targeted by the alteration in miRNA expression. One of the identified adipogenesis-regulated miRNAs, mir-21, showed a positive correlation with obesity in subcutaneous adipose tissue from healthy humans and this merits further investigation to verify the location of mir-21 expression in situ, in adipose tissue.

### MiRNAs regulated by adipogenesis

Fiftyone miRNAs were significantly upregulated and fourteen downregulated during the differentiation of both white and brown preadipocytes. The trend for differences in the degree of miRNA upregulation or downregulation between the white and brown adipocytes was 2-fold. MiR-143 and miR-181a were expressed in both white and brown adipocytes during both developmental stages and showed a trend for a 2-fold enrichment in white adipocytes, similarly to what was found by Walden et al. [16]. However, from this data set, differences in miRNA regulation between white and brown adipocyte population were very modest. 'Muscle-enriched' miRNAs, miR-1 and miR-133a, previously shown by Walden et al. to be modestly yet expressed in brown versus white adipocytes (but not upregulated during their differentiation), were too low in expression to be detected by this microarray study.

Two of the six miRNAs showed a trend for a stronger upregulation during white adipocyte differentiation - miR-24-1* and miR-23b. They belong to a recently identified miR-23b cluster shown to be a molecular switch regulating TGFྞ response of liver stem cells during their differentiation [40], and also to be regulated by bone morphogenetic protein-2 (BMP-2) in mesenchymal stem cells differentiating into adipocytes [41]. The BMP-2 signalling pathway plays a role in white adipocyte lineage determination, as BMP-2 can induce commitment of C3H10T1/2 pluripotent stem cells into white adipocytes [42]. Our results, in combination with these previous observations, suggest that this mechanism might therefore involve miR-24-1 and miR-23b. Another important member of this miR-23b cluster is miR-27b, which has also been shown to play important roles in adipogenesis by impairing differentiation of human adipocytes and targeting PPARγ [43].

However, we found that miR-27b was not robustly expressed in brown or white *primary *adipocytes. Two miRNA, miR-669c and miR-34c, demonstrated a trend for downregulation during the maturation of white but not brown adipocytes. miR-669c has recently been implicated in ageing [44] and correlates with the decline of liver regeneration. The only miRNA that was significantly downregulated in both brown and white adipocytes (yet more so during the brown adipocyte maturation) was let-7e, which belongs to the let-7 family. Let-7 impairs adipogenic differentiation [29], explaining its downregulation during the adipogenic process. Consistent with *in vitro *adipogenesis, which consists of two processes - cell proliferation followed by differentiation - most of the miRNAs regulated by adipogenesis in our system are involved in the regulation of these two processes.

### Evaluation of the similarity to other models through literature study

Ortega et al. examined global miRNA expression profiles during human adipogenesis and whether its pattern significantly differed between cells from obese or lean subjects [14]. The differentiation protocol they used differed significantly from ours. They cultured human preadipocytes and passed them through 3 passages before the differentiation was induced at confluency using a cocktail of human Insulin, Dexamethasone, Isobutylmethyl-xanthine (IBMX), and the PPARγ agonist Rosiglitazone, in the presence of fetal bovine serum. This cocktail of drugs is usually necessary to promote successful differentiation in clonal cell lines. The cells we isolated from fat tissues were plated and the differentiation was induced spontaneously in media supplemented with only insulin and newborn bovine serum, and as such it perhaps provides a more native differentiation context. In Ortega's findings most of the miRNAs showed increased or decreased regulation after 7 days of differentiation. They identified the cluster of miRNAs related to miR-30 (miR-30a, b, c, d and e) as being increased during human white adipocyte maturation and these data are in accordance with our observations [14]. Ortega et al., however, show miR-503 as the most down-regulated miRNA during differentiation of human adipocytes [14], while in our murine data set miR-503 was upregulated during both primary brown and white adipocyte differentiation.. The discrepancy with respect to mir-503 regulation may relate to differential regulation between man and mouse, or differences in the differentiation protocols applied.

Our results are also mostly in agreement with those of Esau et al. [25] who identified a similar expression pattern regarding miR-130b, miR-30c, miR-30a*, miR-191, miR-30d, miR-196, miR-30b, miR-19b, miR-92, miR-138 and miR-100 during differentiation of cultured human adipocytes. However, our results are not in accordance with Esau regarding miR-20, miR-93, miR-103 and miR-107. Esau et al., similarly to Ortega et al., used a cocktail of drugs to induce the differentiation of human preadipocytes. Thus, these discrepancies could again reflect differences between human and mouse adipogenesis or differences in *in vitro *culture conditions. Xie et al. [27] identified miR-143, miR-148a, miR-30c, miR146b as being upregulated during *in vitro *differentiation of 3T3-L1 cells, which is identical to our findings. However, Xie et al. additionally found miR-107 and miR-103 to be upregulated, similarly to Ortega's findings in human adipocytes, but contrary to our results. As the regulation of mir-107 and mir-103 exists in man and mouse, differences in the differentiation protocols applied could explain the discrepancy. One must also consider the time when such miRNA analysis was run and the possibility of alterations in the annotation of both miRNA sequence and chip design.

One example of miRNAs that can be actively involved in cell proliferation is the miR-17-92 cluster, which comprises seven miRNAs (miR-17-5p, miR-17-3p, miR-18, miR-19a, miR-20, miR-19b, and miR-92-1). This miR-17-92 cluster is frequently amplified in B-cell lymphomas and lung cancers, and promotes tumour growth in human and mouse cell models [45,46]. Wang et al. used 3T3-L1 preadipocyte cells to screen miRNA expression over seven timepoints after hormonal induction [28]. They found all five members of the miR-17-92 cluster to be markedly upregulated after hormonal stimulation, peaking at the clonal expansion stage. In our dataset, similarly, all members of the cluster were upregulated, suggesting that this cluster might play a role during both proliferation and differentiation of adipocytes.

### Predicted protein targets and network analysis

To better understand the biological consequences of simultaneous changes in multiple miRNAs we utilised a weighted score ranking analysis method [19], which identifies the protein targets most likely to be regulated by collective changes in miRNA expression. We found the ontologies, transcription regulation and regulation of cellular metabolic processes, were strongly represented in the miRNA target list. We made a provisional assessment of the connectivity of the genes belonging to the ontology category 'SP_PIR_Transcription Regulation' using the literature co-citation network PubGene. Sirt1 was linked directly to 5 other genes from the analysed group. Sirt1, an NAD^+^-dependent deacetylase, belongs to the family of sirtuins, and regulates key aspects of lipid metabolism. Sirt1 plays a role in 3T3-L1 adipogenesis, as Sirt1 protein levels increase during adipogenesis, and overexpression of Sirt1 prevents adipogenesis by inhibiting PPARγ transactivation of adipogenesis-related genes, while knock-down of Sirt1 promotes fat accumulation [47]. Activation of Sirt1 increases the release of FFA in both mature 3T3-L1 adipocytes and in primary white rat adipocytes [47]. Sirt1 also appears to exert an anti-inflammatory effect and improves insulin sensitivity in 3T3-L1 adipocytes by promoting insulin-stimulated glucose uptake [48]. Accordingly, Sirt1 activators improve insulin resistance in ob/ob and diet-induced obese mice, and increase insulin sensitivity in obese, insulin-resistant Zucker rats [49]. Sirt1 genetic variation in humans is related to BMI and risk of obesity [50]. The miRNAs targeting Sirt1 include miR-143, miR-23b miR-34c as well as mir-34a [51,52].

### Adipogenesis-regulated miRNAs and human obesity

Of the miRNAs we described in the murine primary cell cultures, miR-21 and miR-143 were differentially expressed in healthy non-obese persons (BMI <30) versus obese persons (BMI >30). Furthermore, we found a robust positive correlation of mir-21 with BMI in healthy humans. In the ob/ob mouse, which develops obesity and type 2 diabetes-like symptoms, expression of mir-21 in liver is downregulated [53] suggesting that mir-21 may have different roles depending on the cell type. Both miR-21 and miR-143 show an altered expression level in cancer: miR-21 is upregulated 5-fold in colorectal cancer, when compared with adjacent non-tumour tissue, while miR-143 is downregulated 3-fold [54]. In human hepatocellular cancer [55], and in breast tumours [56], miR-21 is also overexpressed, when compared with normal tissue. An anti-miR-21 strategy decreases tumour growth in vivo, possibly by enhancing apoptosis [56]. Cell proliferation is increased by miR-21 overexpression, while it is decreased by an anti-miR-21 strategy, when modest changes in miR-21 expression are achieved (2 to 4-fold changes) [55]. In obese subjects there is an expansion of fat tissue, and while mir-21 is positively correlated to BMI, it is unknown whether mir-21 can induce adipose tissue expansion, or if it is increased as a consequence of adipose tissue expansion or the inclusion of additional cell types within the tissue. Gabriely et al. found that downregulation of mir-21 in glioma cells reduces their invasive potential, probably by relieving mir-21 targeting of metalloprotease inhibitors, allowing metallo proteases to be active [57]. These data thus suggest that mir-21 could be important for tissue expansion, albeit this will require substantial experimental verification.

## Conclusion

We have identified 65 miRNAs regulated during adipogenesis in primary brown and white adipocytes and show that one miRNA, mir-21, correlates positively with human obesity. We also compared our list of adipogenesis-regulated miRNAs with different model systems and find that there are important differences between cell lines or animal models of obesity, and our primary adipocyte profiles. We provide an mRNA and miRNA gene-chip resource for further analysis.

## Competing interests

The authors declare that they have no competing interests.

## Authors' contributions

PK carried out the qPCR and EXIQON gene chip analysis. VG performed the PubGene analysis and data mining. VG and PK drafted the manuscript. NP carried out preparation of murine samples for microarray analysis. IJG performed the miRNA target ranking and Gene Ontology analysis. JT, JN and BC conceived the study, and participated in its design, coordination and microarray analysis. All authors edited the article and all authors read and approved the final manuscript.

## Pre-publication history

The pre-publication history for this paper can be accessed here:

http://www.biomedcentral.com/1472-6823/11/7/prepub

## Supplementary Material

Additional file 1 **Significantly upregulated and downregulated miRNAs during the differentiation of both white and brown preadipocytes combined**. Click here for file

Additional file 2 **Gene ontology analysis of the upregulated and downregulated putative targets of miRNAs**. Click here for file
